# Supplementary material for: Azobenzene as Antimicrobial Molecules
Source: Molecules. 2022 Sep 1;27(17):5643. doi: 10.3390/molecules27175643 (PMC9457709; doi:10.3390/molecules27175643)
Supplement: Supplementary file 1 [file molecules-27-05643-s001.zip › molecules-1878024-supplementary.pdf]

Supplementary Information

Azobenzenes as antimicrobial molecules

Miriam Di Martino [1], Lucia Sessa [1], Martina Di Matteo [1], Barbara Panunzi [2], Stefano Piotto [1,3] and Simona Concilio [1,3,*]

1 Department of Pharmacy, University of Salerno, Via Giovanni Paolo II, 132, 84084 Fisciano, Italy
2 Department of Agriculture, University of Napoli Federico II, 80126 Naples, Italy
3 Bionam Research Center for Biomaterials, University of Salerno, Fisciano, Italy
* Correspondence: sconcilio@unisa.it

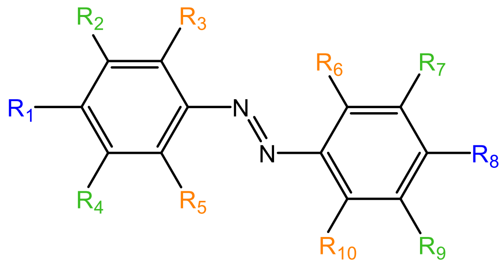

Table S1 – Antimicrobial activities of azobenzene based compounds

R1	R2	R3	R4	R5	R6	R7	R8	R9	R10	Gram–		Gram+		Ref.
														**Minimum inhibitory concentration (MIC)**
hydroxy						ethyl	hydroxy			>60	>60	30	50	*[1]* The Gram+ tested were *S. aureus A170* (on the left) and *L. monocytogenes* (on the right).
hydroxy						allyl	hydroxy			>60	>60	20	25	
hydroxy						propyl	hydroxy			>60	>60	20	25	

hydroxy						methyl	hydroxy	methyl		>60	>60	20	25	The Gram- tested were *Salmonella typhimurium* (on the left) and *Pseudomonas aeruginosa ATCC-27853* (on the right). The values are expressed as MIC$_0$ (µg/mL)
hydroxy						methyl	hydroxy	allyl		>60	>60	25	> 60	
butoxy						methyl	hydroxy	methyl		>128	>128	32	>128	[2] The Gram+ tested were Staphylococcus aureus ATCC 29213 (on the left) and L. monocytogenes (on the right). The Gram- tested were *Pseudomonas aeruginosa PAO1* (on the left) and *Escherichia coli MG1655* (on the right). The values are expressed as MIC$_{100}$ (µg/mL)
isobutoxy						methyl	hydroxy	methyl		>128	>128	128	128	
neopentyloxy						methyl	hydroxy	methyl		>128	>128	>128	>128	
Isopentyloxy						methyl	hydroxy	methyl		>128	>128	4	8	
2-Ethylhexyloxy						methyl	hydroxy	methyl		>128	>128	12	48	
						methyl	hydroxy	methyl		>128	>128	>128	>128	
isobutoxy					hydroxy		hydroxy			>128	>128	>128	96	
neopentyloxy					hydroxy		hydroxy			>128	>128	4	8	
isopentyloxy					hydroxy		hydroxy			>128	>128	4	8	
2-Ethylhexyloxy					hydroxy		hydroxy			>128	>128	16	16	
hydroxy					hydroxy		hydroxy			>128	>128	>128	>128	
methyl					hydroxy		hydroxy			>128	>128	>32	>32	
methoxy					hydroxy		hydroxy			>128	>128	16	16	
	hydroxy					methyl	hydroxy	methyl		>35		30		[3] The Gram+ tested was
						methyl	hydroxy	methyl		>35		7		

methoxy						methyl	hydroxy	methyl		>35		>35		Staphylococcus aureus A170. The Gram-teste was *P. aeruginosa ATCC-27853*. The values are expressed as MIC_{50} (μg/mL)
methyl						methyl	hydroxy	methyl		>35		>35		
hydroxy										>35		25		
hydroxy							Methoxy			>35		25		
hydroxy							methyl			>35		7		
hexyl							N,N,N-trimethylethanaminium			64	>64	16	4	[4] MIC values (μg/mL) against the studied Gram-positive and Gram-negative strains. Gram-: *E. coli ATCC 25922* on the left and *P. aeruginosa ATCC 47085* on the right. Gram+: *S. aureus ATCC 29213* on the left and *S. aureus ATCC 43300* on the right.
heptyl							N,N,N-trimethylethanaminium			8 trans	>64 trans	1 trans	1 trans	
heptyl							N,N,N-trimethylethanaminium			16 cis	- cis	4 cis	2 cis	
decyl							N,N,N-trimethylethanaminium			>64	>64	8	8	
hexyl							tobramycin			64	32	64	64	
heptyl							tobramycin			64	16	16	8	
heptyl							tobramycin			32	4	4	8	
decyl							tobramycin			>64	>64	64	>64	
methoxy					quinolone					8	16	*NA*	*NA*	*[5]* Antibacterial activity was determined before (on
	methyl				quinolone					>64	16	*16*	2	

	methyl		methyl		quinolone					>64	>64	*NA*	*NA*	the left) and after irradiation (on the right) at 365 nm on *Escherichia coli CS1562*. The compounds 2 was tested against the Gram+ *M. luteus*.
methoxy	methyl	methyl		methyl	quinolone					>64	32	*NA*	*NA*	
	methyl			methoxy	quinolone					16	16	*NA*	*NA*	
methoxy	fluoride				quinolone					>64	64	*NA*	*NA*	
methoxy	fluoride		fluoride		quinolone					>64	32	*NA*	*NA*	
	fluoride			methoxy	quinolone					16	16	*NA*	*NA*	
methoxy	methoxy				quinolone					64	64	*NA*	*NA*	
	bromide						hydroxy	4-chloroaniline		3.02	1.51	3.02	3.02	[6] Antimicrobial activity of diazenyl schiff bases expressed as MIC values (µg/mL). Gram-: *E. coli MTCC 1652* (on the left) and *P. aeruginosa MTCC 1688* (on the right). Gram+: *S. aureus MTCC 2901* (on the left) and *B. subtilis MTCC 2063* (on the right).
nitro		chloride					hydroxy	2-fluoroaniline		3.14	1.57	6.28	3.14	
	bromide						hydroxy	4-fluoro-2-methoxyaniline		1.37	1.37	1.37	2.75	
		bromide					hydroxy	4-nitroaniline		1.47	1.47	1.47	2.94	
chloride	nitro						hydroxy	2-methylaniline		3.17	1.59	3.17	3.17	
chloride							hydroxy	2,4-dimethylaniline		1.72	1.72	1.72	3.44	
	chloride						hydroxy	2-methyl-4-chloroaniline		0.86	1.72	1.72	1.72	
			chloride				hydroxy	4-(methylthio)aniline		1.5	1.5	1.5	3	
		chloride	chloride				hydroxy	2,4-dimethylaniline		1.57	1.57	0.78	3.14	

	chloride						hydroxy	2,4-dimethylaniline		1.72	3.44	3.44	3.44	
methyl		methyl					hydroxy	2,5-dichloroaniline		1.57	1.57	0.78	3.14	
methyl		methyl					hydroxy	4-fluoroaniline		0.9	0.9	0.9	3.6	
		fluoride					hydroxy	2,6-dimethylaniline		3.6	3.6	3.6	3.6	
	bromide						hydroxy	2,5-dichloroaniline		1.39	1.39	1.39	2.78	
	bromide						hydroxy	2-chloro-4-nitroaniline		1.36	1.36	1.36	2.72	
nitro		chloride					hydroxy	3-methoxyaniline		3.05	1.52	1.52	3.05	
nitro		chloride					hydroxy	4-fluoroaniline		3.14	3.14	3.14	3.14	
nitro		chloride					hydroxy	2,6-dimethylaniline		3.06	3.06	3.06	3.06	
		fluoride					hydroxy	2,5-dichloroaniline		3.22	3.22	3.22	3.22	
		fluoride					hydroxy	2,4-dimethylaniline		1.8	1.8	0.9	3.6	
		chloride	chloride				hydroxy	2-methoxyaniline		0.78	0.78	0.78	3.13	
		chloride	chloride				hydroxy	3-bromoaniline		0.69	1.39	0.69	2.78	
		chloride	chloride				hydroxy	4-fluoroaniline		1.61	1.61	1.61	3.22	
methyl		methyl					hydroxy	3-bromoaniline		0.76	1.53	0.76	3.06	

bromide							hydroxy	2-methylaniline		1.53	1.53	1.53	3.06	
methyl							hydroxy	N-p-tolylmethanimine		500		250		[7] The MIC (mg/mL) of each azo Schiff base was expressed as mg/mL and was calculated for *P. aeruginosa* and *S. aureus*.
methyl							hydroxy	N-(4-nitrophenyl)methanimine		50		50		
nitro							hydroxy	2,3-dihydro-1H-perimidine		6.25	1.56	3.13		[8] The in vitro antibacterial activities of the synthesized azo fused pyrimidines were performed against *P. aeruginosa (PA01)* (on the left), *E. coli (ATCC 25922)* (on the right) and *B. cereus (ATCC 11778)*. The values are expressed as MIC (µg/mL).
	nitro						hydroxy	2,3-dihydro-1H-perimidine		12.5	3.13	6.25		
N-(4,6-dimethylpyrimidin-2-yl)sulfonic amide							N,N-dimethylamine			50	12.5	3.12		[9] The organisms used for the antimicrobia

N-(4,6-dimethylpyrimidin-2-yl)sulfonic amide							N,N-diethylamine			50	25	0.8	l investigation are *E. coli* (on the left), *P. aeruginosa* (on the right) and *E. faecalis*. The values are expressed as MIC (mg/mL).
N-(4,6-dimethylpyrimidin-2-yl)sulfonic amide							aniline			25	25	0.4	
hydroxy					bromide					111.7		159.5	[10] The antibacterial activity of the compounds was screened against *E. coli ATCC 25922* and *S. aureus S48/81*. Here we report the MIC values (ppm).
hydroxy						bromide				118.3		140.9	
hydroxy					iodide					130.5		164.2	
hydroxy						iodide				145.1		155.3	
aspirine					bromide					>220		>220	
aspirine						bromide				>220		>220	
aspirine					iodide					>220		>220	
aspirine						iodide				>220		>220	
hydroxy			carboxyl		bromide					89		89	
hydroxy			carboxyl			bromide				7594		80	
hydroxy			carboxyl		iodide					75		64	
hydroxy			carboxyl			iodide				85		84	
													**Inhibition zones (mm)**
hydroxy				hydroxy	nitro					NA		27	[11] Activity toward Gram+ was tested on *S. aureus* strain NCTC 6571 using 0.4 mg/mL
hydroxy				hydroxy		nitro						39	
hydroxy				hydroxy			nitro					35	

													concentration
hydroxy							decyloxy			10	10	Inactive	*[12]* The Gram- tested were *E. coli (*on the left) and *Salmonella Typhimurium* (on the right). Activity toward Gram+ was tested on *S. aureus* using 1 mg/mL concentration.
hydroxy			fluoride				decyloxy			Inactive	Inactive	Inactive	
hydroxy				fluoride			decyloxy			Inactive	Inactive	11	
4-bromohexyloxy							decyloxy			13	12	14	
4-bromohexyloxy			fluoride				decyloxy			14	12	11	
decyloxy							N-(hexyl-6-oxy)-N'-decyl imidazole			10	10	Inactive	
decyloxy							N-(hexyl-6-oxy)-N'-dodecyl imidazole			11	10	Inactive	
decyloxy							N-(hexyl-6-oxy)-N'-tetradecyl imidazole			10	10	Inactive	
decyloxy							N-(hexyl-6-oxy)-N'-hexadecyl imidazole			Inactive	Inactive	10	
decyloxy							N-(hexyl-6-oxy)-N'-octadecyl imidazole			Inactive	64	11	
N-(pyrimidin-2-yl)sulfonic amide						formyl	hydroxy	methoxy		45		49	[13] The antimicrobial activity of compounds was tested against *E. coli (ATTC 25922)* and *S. aureus*
N-(pyrimidin-2-yl)sulfonic amide						(4-Bromophenyl) iminomethyl	hydroxy	methoxy		25		22	

N-(pyrimidin-2-yl)sulfonic amide						(2-Chlorophenyl) iminomethyl	hydroxy	methoxy		21		25		*(ATCC 25923)*. Here we report the inhibition zone (mm) of all compounds against bacteria at a concentration of 30 mg/ml.
N-(pyrimidin-2-yl)sulfonic amide						3-Chloro-2-methylphenyl) iminomethyl	hydroxy	methoxy		22		30		
N-(pyrimidin-2-yl)sulfonic amide						4-(methyleneamino)-N-(pyrimidin-2-yl)benzenesulfonamide	hydroxy	methoxy		20		20		
N-(pyrimidin-2-yl)sulfonic amide						(2,4-Dimethylphenyl) iminomethyl	hydroxy	methoxy		30		23		
N-(pyrimidin-2-yl)sulfonic amide						(2-hydroxyphenyl) iminomethyl	hydroxy	methoxy		22		25		
		hydroxy	isopropyl				chloride			16	18	14	13	[14] The Gram– tested were *E. coli ATCC 11229* (on the left) and *Enterobacter cloacae ATCC13047 D* (on the right). The Gram+ tested were *S. aureus ATCC25923* (on the left) and *M. luteus NRLL*
		hydroxy	isopropyl		chloride		chloride			not determined	19	17	24	
		hydroxy	sec-butyl				chloride			not determined	12	13	12	
		hydroxy	sec-butyl		chloride		chloride			14	20	12	20	

														B-4375 (on the right)

References

1. Piotto, S., et al., *Small azobenzene derivatives active against bacteria and fungi.* European journal of medicinal chemistry, 2013. **68**: p. 178-184.
2. Piotto, S., et al., *Synthesis and antimicrobial studies of new antibacterial azo-compounds active against staphylococcus aureus and listeria monocytogenes.* Molecules, 2017. **22**(8): p. 1372.
3. Concilio, S., et al., *Structure modification of an active azo-compound as a route to new antimicrobial compounds.* Molecules, 2017. **22**(6): p. 875.
4. Salta, J., et al., *Tuning the Effects of Bacterial Membrane Permeability through Photo-Isomerization of Antimicrobial Cationic Amphiphiles.* Chemistry–A European Journal, 2017. **23**(52): p. 12724-12728.
5. Velema, W.A., et al., *Optical control of antibacterial activity.* Nature chemistry, 2013. **5**(11): p. 924-928.
6. Kaur, H., et al., *Diazenyl schiff bases: synthesis, spectral analysis, antimicrobial studies and cytotoxic activity on human colorectal carcinoma cell line (HCT-116).* Arabian Journal of Chemistry, 2020. **13**(1): p. 377-392.
7. Mkpenie, V.N., E.E. Essien, and I.V. Mkpenie, *Antimicrobial activity of azo-schiff bases derived from salicylaldehyde and para-substituted aniline.* World J. Pharm. Res., 2015. **4**(12): p. 52-60.
8. Nagasundaram, N., et al., *Synthesis, characterization and biological evaluation of novel azo fused 2, 3-dihydro-1H-perimidine derivatives: In vitro antibacterial, antibiofilm, anti-quorum sensing, DFT, in silico ADME and Molecular docking studies.* Journal of Molecular Structure, 2022. **1248**: p. 131437.
9. Keshavayya, J., *Synthesis, structural investigations and in vitro biological evaluation of N, N-dimethyl aniline derivatives based azo dyes as potential pharmacological agents.* Journal of Molecular Structure, 2019. **1186**: p. 404-412.
10. Ngaini, Z. and N.A. Mortadza, *Synthesis of halogenated azo-aspirin analogues from natural product derivatives as the potential antibacterial agents.* Natural product research, 2019. **33**(24): p. 3507-3514.
11. Ali, H.M., S.Q. Badr, and M.F.H. Al-Kinani. *DNA Binding three Azo Dyes as new Antibiotics*. in *IOP Conference Series: Materials Science and Engineering*. 2019. IOP Publishing.
12. Babamale, H.F., et al., *Synthesis and characterization of azobenzene derivatives and azobenzene-imidazolium conjugates with selective antimicrobial potential.* Journal of Molecular Structure, 2021. **1232**: p. 130049.
13. Jadou, B.K., A.J. Hameed, and A.Z. Al-Rubaie, *Synthesis, Antimicrobial, Antioxidant and Structural Studies of Some New Sulfa Drug Containing an Azo-azomethine Group.* Egyptian Journal of Chemistry, 2021. **64**(2): p. 751-759.
14. Bal, S., et al., *Synthesis, thermal stability, electronic features, and antimicrobial activity of phenolic azo dyes and their Ni (II) and Cu (II) complexes.* Chemical Papers, 2014. **68**(3): p. 352-361.
